# Listerin

**Published:** 1897-07

**Authors:** W. C. Barrett


ARTICLE VIII.

### LISTERIN.

BY DR. W. C. BARRETT.

The more I use listerin the better I like it. I am now prescribing it as a daily wash for the mouth, using it with the brush in full strength in those cases in which there seems an unusual predisposition to decay of the teeth.

I use it almost invariably after any surgical operation in the mouth as an antiseptic wash, 1 part to 10 to 20 parts of water.

In gingivitis, arising from a want of the care of the teeth, I inject it under the gums freely, and prescribe it as a daily mouth wash, using listerin 1 part, to 5 to 10 parts of water.

In the treatment of septic roots of teeth, I have seen the happiest effects from the use of listerin, forcing a pledget of cotton into the pulp cavity, after carrying a few threads of cotton wet with listerin into the root canals, and sealing the whole in the cavity with gutta-percha or wax.

In abscesses, I have frequently injected it with a syringe quite through the fistulous track, with the result of a complete cure by from one to three such treatments.

In diseases of the maxillary sinus, listerin is now my principal reliance. An opening having been secured, it is injected into the cavity by means of a spray syringe, diluting it with from 20 to 30 parts of water.

In pyorrhea, after the surgical treatment and the removal of deposits, I have found nothing better than listerin for daily use with a soft brush, diluting it with equal parts of water.

In pharyngitis, accompanying inflamed condition of the oral cavity, I use it as a spray on the pharyngeal tissues. If the patient be an adult, I frequently use it thus in full strength.

After nearly all protracted operations in the mouth, I offer as a gargle and mouth wash 20 to 30 drops in a half tumbler of water, and find it leaves the mouth in a delightfully cool and pleasant condition, promotes the healthy granulation of lacerated or bruised tissues, and offers a sure prevention to any possible septic infection. So general is its use by me, that a large proportion of my patients leave the office with the pleasant taste of listerin in their mouth. Thus their last impression is one of purity and cleanliness, and of careful attention on the part of their dentist.—*Items of Interest.*

---

ARTICLE IX.

